# Genome Wide Association Study and Genomic Selection of Amino Acid Concentrations in Soybean Seeds

**DOI:** 10.3389/fpls.2019.01445

**Published:** 2019-11-15

**Authors:** Jun Qin, Ainong Shi, Qijian Song, Song Li, Fengmin Wang, Yinghao Cao, Waltram Ravelombola, Qi Song, Chunyan Yang, Mengchen Zhang

**Affiliations:** ^1^National Soybean Improvement Center Shijiazhuang Sub-Center, North China Key Laboratory of Biology and Genetic Improvement of Soybean, Ministry of Agriculture, Laboratory of Crop Genetics and Breeding of Hebei, Cereal & Oil Crop Institute, Hebei Academy of Agricultural and Forestry Sciences, Shijiazhuang, China; ^2^Department of Horticulture, University of Arkansas, Fayetteville, AR, United States; ^3^Soybean Genomics and Improvement Lab, USDA-ARS, Beltsville, MD, United States; ^4^Crop and Soil Environmental Science, Virginia Tech, Blacksburg, VA, United States; ^5^Bioinformatics Center, Allife Medical Science and Technology Co., Ltd, Beijing, China

**Keywords:** *Glycine max*, genome-wide association study, genomic selection, genotyping by sequencing, amino acid concentration, single nucleotide polymorphism

## Abstract

Soybean is a major source of protein for human consumption and animal feed. Releasing new cultivars with high nutritional value is one of the major goals in soybean breeding. To achieve this goal, genome-wide association studies of seed amino acid contents were conducted based on 249 soybean accessions from China, US, Japan, and South Korea. The accessions were evaluated for 15 amino acids and genotyped by sequencing. Significant genetic variation was observed for amino acids among the accessions. Among the 231 single nucleotide polymorphisms (SNPs) significantly associated with variations in amino acid contents, fifteen SNPs localized near 14 candidate genes involving in amino acid metabolism. The amino acids were classified into two groups with five in one group and seven amino acids in the other. Correlation coefficients among the amino acids within each group were high and positive, but the correlation coefficients of amino acids between the two groups were negative. Twenty-five SNP markers associated with multiple amino acids can be used to simultaneously improve multi-amino acid concentration in soybean. Genomic selection analysis of amino acid concentration showed that selection efficiency of amino acids based on the markers significantly associated with all 15 amino acids was higher than that based on random markers or markers only associated with individual amino acid. The identified markers could facilitate selection of soybean varieties with improved seed quality.

## Introduction

Soybean [*Glycine max* (L.) Merr.] is a major source of protein for humans and livestock in the world. For the past several decades, soybean meal has been the leading protein feed source for the animal and poultry production operations because of its high concentration of protein. Poultry and livestock industries use about 68 and 77% of the soybean meal consumed in the European Union and United States, respectively^1^^,^^2^. A major function of proteins in nutrition is to supply adequate amounts of required amino acids (Friedman and Brandon, 2001). Thus, genetic improvement of amino acid composition and balance is an important goal in soybean breeding. Developing new molecular markers for marker assisted selection (MAS) and genomic selection (GS) of amino acid composition in soybean will help to achieve this goal.

Quantitative trait loci (QTL) mapping of amino acids have been reported in soybean. Panthee et al. (2006) identified 32 simple sequence repeat (SSR) markers associated with 16 amino acids in soybean seeds based on 101 F6-derived recombinant inbred lines (RIL) from a cross of N87-984-16 × TN93-99. Fallen et al. (2013) reported ten QTLs associated with 17 amino acids and three genomic regions on chromosome 13 (4.89, 21.51, 40.69 cM) controlled multiple amino acids in 282 F5:9 RILs derived from a cross of Essex × Williams 82. As a sole dietary source of protein, soybean is deficient in lysine (Lys), threonine (Thr), methionine (Met), and cysteine (Cys) for poultry and swine. Warrington et al. (2015) conducted QTL analysis for the four amino acids in the Benning × Danbaekkong soybean population with 98 SSRs and 323 single nucleotide polymorphism (SNP) markers, and detected two QTLs on chr 8 and 20 for Lys; three on chr 9, 17, and 20 for Thr; four on Chr 6, 9, 10, and 20 for Met; and one on chr 10 for Cys (Van Warrington, 2011; Warrington et al., 2015). Khandaker et al. (2015) analyzed MD96-5722” × “Spencer” RIL population and identified 13 QTLs associated with amino acids. However, reports of genetic diversity of amino acids and mapping of QTLs controlling amino acid in soybean germplasm are limited.

Because SSR, SNPs, and indels are abundant in plants and can be assayed with high-throughput technology, the markers have been widely used for genetic linkage mapping, association studies, diversity analysis, and tagging of genes controlling important traits (Liang et al., 2010; Lehne et al., 2011; Li et al., 2014; Shi et al., 2016; Taranto et al., 2016; Zatybekov et al., 2017; Qin et al., 2017a; Qin et al., 2017b; Chang et al., 2018). Genotyping by sequencing (GBS) takes advantage of the next-generation sequencing platforms and utilizes a highly-multiplexed system to assay DNA variants from reduced representation DNA libraries of plant materials (Elshire et al., 2011; Sonah et al., 2013). As a cost-effective technique, GBS has been successfully used in implementing genome wide association study (GWAS), genomic diversity study, genetic linkage analysis, molecular marker discovery and GS in plant breeding programs (Heslot et al., 2013; He et al., 2014; Qin et al., 2016; Shi et al., 2017).

With the decreased genotyping cost and improved statistical methods, GWAS and GS offer new approaches for genetic improvement of complex traits in crop species (Bernardo and Yu, 2007; Li et al., 2013; Morris et al., 2013; Yano et al., 2016; Zhang et al., 2017). GWAS is one of the powerful tools to overcome limitations in traditional QTL mapping (Luo et al., 2019). To date, it has been used to identify molecular markers for a broad range of complex traits in different plant species including Arabidopsis (Angelovici et al., 2017), wheat (Peng et al., 2018), maize (Li et al., 2013; Deng et al., 2017), rice (Huang et al., 2010; Yano et al., 2016), soybean (Fang et al., 2017); sorghum (Morris et al., 2013). In soybean research, GWAS were used in agronomic traits (Zatybekov et al., 2017; Chang et al., 2018), seed quality (Zhang et al., 2018), seed traits (Xia et al., 2018), phosphorus efficiency (Lü et al., 2018), disease resistance (Qin et al., 2017b; Hanson et al., 2018) etc. As soybean is globally cultivated primarily for its protein and oil, and soybean protein is a complete protein as it contains all the essential amino acid that are required for human health. Numerous studies have reported on the QTL mapping and GWAS for protein (Li et al., 2018; Li et al., 2019). GS is to select desired individual within a population based on genomic estimated breeding values (GEBVs) (Hayes et al., 2009), GS has been shown more efficient than the traditional MAS for the improvement of traits controlled by QTL with minor effects (Bernardo and Yu, 2007; Heffner et al., 2009; Shikha et al., 2017; Zhang et al., 2017). GS has been applied to various agronomic traits and disease resistance in maize (Bernardo, 1996; Piepho, 2009; Albrecht et al., 2011; Technow et al., 2013; Shikha et al., 2017), rice (Onogi et al., 2015; Spindel et al., 2015; Duhnen et al., 2017), soybean (Jarquin et al., 2016; Xavier et al., 2016), and wheat (Heffner et al., 2011; Rutkoski et al., 2011; Poland et al., 2012; Battenfield et al., 2016), etc. Previous studies reported the efficiency of GS prediction by cross-validation approach (Dawson et al., 2013; Michel et al., 2016) and suggested that the size of the training population was critical (Xavier et al., 2016). Zhang et al. (2018) conducted GWAS for seed composition, including protein, oil, fatty acids, and amino acids, using 313 diverse soybean germplasm accessions genotyped with a high-density SNP array of the Illumina Infinium SoySNP50K BeadChip (Song et al., 2013). After filtered, a total of 31,850 SNPs with minor allele frequency (MAF) ≥5% were used for GWAS in their analysis and 87 chromosomal regions were identified to be associated with seed composition, explaining 8–89% of genetic variances.

However, little GWAS and no GS for amino acid concentrations in soybean has been reported so far. The main objectives of this study were to (1) evaluate amino acid compositions in soybean germplasm from China, Korea, Japan and U.S. (2) identify SNP markers associated with amino acid concentrations of soybean *via* GWAS, and (3) explore efficiency of GS for amino acids in soybean breeding. The newly identified markers are anticipated to facilitate MAS and GS of nutritional traits in soybean, and the soybean accessions with high concentrations of amino acids will be potential parents for soybean breeding.

## Materials and Methods

### Panel for Genome-Wide Association Analysis and Genomic Selection

The panel with a total of 249 soybean accessions was chosen for this study (**Supplementary Table 1**). These accessions were collected from China, United States, South Korea, and Japan with 169 (67.9% out of 249), 75 (30.1%), 3 (1.2%), and 2 (0.8%) accessions, respectively (**Supplementary Table 1**).

### DNA Extraction, GBS, and SNP Discovery

Genomic DNA was extracted from freeze-dried fresh leaves of soybean plants using the CTAB (hexadecyltrimethyl ammonium bromide) method (Kisha et al., 1997). DNA library was prepared using the fragment digested by restriction enzyme ApeKI following the GBS protocol described by Elshire et al. (2011) and DNA sequencing was performed using GBS method (Elshire et al., 2011; Sonah et al., 2013). The 90 bp pair-end sequencing was obtained from each soybean genotype at the Institute of Genetics and Developmental Biology, Chinese Academy of Sciences, Beijing, China. The GBS dataset contained 3.26 M short-reads or 283.74 Mbp of sequence for each accession. The short reads were aligned to soybean whole genome sequence (Wm82.a1.v1)^3^^,^^4^ using SOAPaligner/soap2 and SOAPsnp v. 1.05 was used for SNP calling (Li et al., 2009; Li, 2011).

Approximately a half million SNPs were discovered from the 249 soybean germplasm accessions. SNPs were eliminated if MAF was less than 5%, or missing and ambiguous alleles larger than 15%. After filtering, 23,279 SNPs remained for genetic diversity and association analyses.

### Amino Acid Content Determination and Phenotypic Data Analysis

Soybean germplasm was grown at three locations, Shijiazhuang (114°83′E, 38°03′N), Cangzhou (116°7′E, 38°03′N), and Handan (114°48′E, 36°62′N) in Hebei province in a randomized complete block design with three replications in June 2012. Each plot consisted of six rows with a row length of three meters and raw space of 50 cm in all trials. The density was 225,000 plants per ha. The soil at Shijiazhuang was cinnamon. The organic matter, available P and available K concentration were 1.74% 29.9 mg/kg, 94.3 mg/kg, respectively. The soil at Cangzhou was light loamy. The organic matter, available P and available K concentration were 1.0–1.2%, 15 mg/kg, and 100 mg/kg, respectively. The soil at Handan was fluviatile loamy and the organic matter, available P and available K concentration were 1.6%, 19.3 mg/kg, 156.2 mg/kg, respectively. The plots were irrigated once at seed-filling stage. Plants were harvested after 95% leaves had fallen off. Ten plants were randomly chosen from the middle of a plot for seed traits analysis.

A total of 15 amino acids, Ala, Arg, Asp, Glu, Gly, His, Ile, Leu, Lys, Phe, Pro, Ser, Thr, Tyr, and Val in soybean seeds were measured by Biochrom 30 amino acid analyzer (Biochrom Ltd, Cambridge, UK) using the acid hydrolysis method (Davies and Thomas, 1973; Tsugita and Scheffler, 1982). Analysis was carried out by ion exchange chromatography under the experimental conditions recommended for protein hydrolysates. Each sample containing 0.1 g soybean seed powder was acid hydrolyzed with 10 ml of 6 N HCl at 110°C for 22 h in a 15 ml vacuum-sealed glass tube. The top hydrolysate in the tube was filtered into another 50 ml tube, and water was added to the tube. A total of 1 ml liquid from the 50 ml tube was transferred to a 1.5 ml tube and dried at 55°C, re-dissolved with 1 ml loading buffer and measured in the analyzer. The amino acid composition was calculated from the standard area obtained from the integrator and expressed as a percentage of the total weight.

Statistical analyses of the 15 amino acids were performed by JMP Genomics 7 (SAS Institute, Cary, NC, USA)^5^ (Sall et al., 2012). The mean, range, standard deviation (SD), standard error (SE) and coefficient of variation (CV) were estimated for each amino acid concentration using ‘Tabulate’; the distributions of amino acid concentrations were drawn using ‘Distribution’ in JMP Genomics 7.

### Population Structure, Genetic Diversity, and Association Analysis

STRUCTURE, a program that uses Bayesian method to analyze multi-loci data in population genetics (Pritchard et al., 2000)^6^, was used to analyze population structure and to create Q-matrix for association analysis. We used the default parameters of STRUCTURE 2.0 software: Admixture Model; Allele Frequencies Correlated; and Compute Probability of the Data (Kaeuffer et al., 2007). The number of subpopulation (K) was assumed to be between 1 and 12. Thus, each K was run 10 times, the Markov Chain Monte Carlo (MCMC) length of the burn-in period was 20,000 and the number of MCMC iterations after the burn-in was 20,000. For each simulated K, the statistical value delta K was calculated using the formula described by Evanno et al. (2005). The optimal K was determined using STRUCTURE HARVESTER^7^ (Earl, 2012). After optimal K was determined, a Q-matrix was obtained and used in TASSEL 5 (Bradbury et al., 2007) for association analysis. Each soybean accession was then assigned to a cluster (Q) based on the probability that the genotype belonged to that cluster. The cut-off probability for the assignment to a cluster was 0.5. Based on the optimum K, a bar plot with ‘Sort by Q’ was obtained to visualize the population structure among the 249 accessions. Genetic diversity was also assessed and the phylogenic tree was drawn using MEGA 6 (Tamura et al., 2013) based on the Maximum Likelihood (ML) tree method (Shi et al., 2016) with the following parameters. Test of phylogeny: bootstrap method with No. of Bootstrap replications 500; Model/Method: General Time Reversible model, Rates among Sites: Gamma distributed with Invariant sites (G/I), Number of Discrete Gamma Categories: 6, Gaps/Missing Data Treatment: Use all sites, ML Heuristic Method: Subtree-Pruning-Regrafting-Ex-tensive (SPR level 5), Initial Tree for ML: Make initial tree automatically (Neighbor Joining), and Branch Swap Filter: Moderate. The population structure and the cluster information were imported to MEGA 6 for combined analysis of genetic diversity. For sub-tree of each Q (cluster), the shape of ‘Node/Subtree Marker’ and the ‘Branch Line’ was drawn using the same color scheme of the STRUCTURE analysis.

Association mapping for the 15 amino acids was conducted separately based on the mixed linear model (MLM-Q+K) in TASSEL 5^8^ (Bradbury et al., 2007) The SNP markers were considered significantly associated with amino acids if logarithm of the odds (LOD) value ≥3.0 based on MLM-Q+K models.

### Linkage Disequilibrium Analysis and SNP-Based Haplotype Blocks

TASSEL 5.0 (Bradbury et al., 2007) was used to calculate the linkage disequilibrium (LD) (r2) for all pairwise loci within a window of 1MB of each chromosome. Haplotype blocks (HAP) were constructed in Haploview (Barrett et al., 2004) with a cutoff of 1% (Contreras-Soto et al., 2017). The LD (r2) for all marker pairs was performed using the R script LDit^9^.

### Candidate Gene Selection

Two databases including the annotations for genes at Soybase at www.soybase.org/dlpages/ ^10^ and the plant metabolic network (PMN) database^11^, were used for searching candidate genes related to amino acids in soybean.

Currently, three Williams 82 genome sequence assemblies are available at Soybase (Glyma1.1, and Glyma 2.0)^10^. However, we used Glyma1.1 as the reference because the SNP data were provided by Institute of Genetics and Developmental Biology, Chinese Academy of Sciences, at the time, Glyma1.1 was the best assembly available. We downloaded gene annotation of Glyma1.1 from Soybase and the corresponding gene positions in the Glyma 2.0 were obtained from https://www.soybase.org/correspondence/index.php^12^. For each SNP significantly associated with amino acids, we searched candidate genes within 10 kb of the SNP position. We also downloaded gene annotation from PMN for candidate gene discovery, because the metabolic pathway in PMN is updated with newer version of the genome (Phytozome v12: Gmax_275_Wm82.a2.v1.protein.fa).

### Genomic Selection

#### Method 1: Ridge Regression Best Linear Unbiased Prediction

Ridge regression best linear unbiased prediction (RR-BLUP) was used to predict genomic estimated breeding value (GEBV) in GS and performed in the rrBLUP package (Endelman, 2011) with the R software Version 3.5.0 (Thuiller et al., 2009). The rr-BLUP is an effective and accurate prediction method as demonstrated in a wide range of traits and crops (Heslot et al., 2012; Jarquín et al., 2014; Lipka et al., 2014; Zhang et al., 2016).

We used 4:1 size ratio of training set and validation set randomly selected from the 249 accessions, which is a four-fold cross-validation, and repeated 100 times. Each training population subset consisting of 199 accessions was randomly selected from the association panel, and the remaining 50 accessions as the validation set (Resende et al., 2012; Shikha et al., 2017).

Two sets of SNPs were used to predict GEBV for each amino acid concentration in each accession: (1) all 23,279 high quality SNPs from GBS, and (2) all 231 SNP markers associated with 15 amino acid concentrations with LOD ≥3.0 from GWAS. In addition, we predicted GEBV for each amino acid concentration based on the SNP markers associated with the amino acid.

The prediction accuracy was estimated using the average Pearson’s correlation coefficient (r) between the GEBVs and observed values for each amino acid concentration in the validation set (Zhang et al., 2010; Resende et al., 2012; Shikha et al., 2017). The training and validation sets were randomly created 100 times and the r value was estimated each time. The average r value was calculated for each amino acid. The r value indicates the prediction accuracy and the selection efficiency of GS.

#### Method 2: Genomic Best Linear Unbiased Prediction

GS was also performed with the genomic best linear unbiased prediction (gBLUP) and the method was extended to compressed best linear unbiased prediction (cBLUP) by using the Compressed Mixed Linear Model (CMLM) approach in GAPIT (Lipka et al., 2012; Tang et al., 2016; www.zzlab.net/GAPIT/gapit_help_document.pdf). In order to conduct a four-fold cross-validation for estimating prediction efficiency, we randomly selected 199 accessions as the training set and the remaining 50 accessions as the validation set to predict GEBV for each accession. GEBV was calculated using the cBLUP in GAPIT using the SNP markers which were associated with the 15 amino acid concentrations with LOD ≥3.0 from GWAS. The Pearson’s correlation coefficient (r) between GEBV and observed value of the amino acid concentrations in both training and validation sets were calculated based on the 249 accessions. A total of 100 replications were used to calculate the r values and the average r value for each amino acid was used as the indicator of prediction accuracy.

## Results

### Phenotypic Variation and Association of Amino Acids in Soybean Seeds

The concentration of 15 amino acids, Ala, Arg, Asp, Glu, Gly, His, Ile, Leu, Lys, Phe, Pro, Ser, Thr, Tyr, and Val varied widely among the 249 accessions (**Supplementary Table 2**). Concentration distribution of all amino acids except for Val, Ile and Gly in the accessions was near normal, indicating the amino acids are complex traits (**Supplementary Figure 1**). Glu and Asp were the main components of soybean seeds, which consisted of 20.1% and 13.3% of the total 15 amino acids, respectively. Glu had the highest concentration (74.42 ppm) among the 15 amino acids, followed by Asp (49.15 ppm). Two to five times of difference were observed between the accessions with the lowest and the highest concentration of Arg, Gly, Ile, Leu, Pro, Thr, and Val (**Supplementary Table 2**). The large variations of the amino acids were also indicated by the high CV values (**Supplementary Table 2**).

Most of the correlation coefficients among the 15 amino acids were greater than the threshold of 0.124 at P = 0.05 significant level (**Table 1**). Significant and negative coefficients were also observed between Asp and Ile, Asp and Val, Ile and Gly, Ile and Ser, etc. (**Table 1**). Based on the correlation coefficient values, the 15 amino acids except for Arg, His, and Pro could be divided into two groups (**Table 1**). Group one consisted of five amino acids: Ala, Asp, Glu, Gly and Ser, their pairwise correlation coefficients were greater than 0.75 except for the pair between Glu and Gly (r = 0.6) (**Table 1**). Group two contained seven amino acids: Ile, Leu, Lys, Phe, Thr, Tye, and Val with r values greater than 0.48 for all pairs. However, most correlation coefficients of amino acids between the two groups were negative (**Table 1**). Since the content of amino acids within each group were all significantly and highly correlated, they could have practical application in breeding program, e.g. breeders don’t need to improve amino acid individually, they can simultaneously improve multiple amino acids within the same group.

**Table 1:** Correlation coefficients among 15 amino acid concentrations in soybean seeds.

		Group 1						Group 2						Arg	His	Pro
		Ala	Asp	Glu	Gly	Ser	Ile	Leu	Lys	Phe	Thr	Tyr	Val			
Group 1	Ala	**1**														
	Asp	0.849*	**1**													
	Glu	0.752*	**0.763**	**1**												
	Gly	**0.785**	**0.846**	**0.600**	**1**											
	Ser	**0.797**	**0.927**	**0.786**	**0.759**	**1**										
Group 2	Ile	-0.392	-0.627	-0.262	-0.713	-0.627	**1**									
	Leu	0.015	-0.236	0.114	-0.350	-0.194	**0.776**	**1**								
	Lys	0.117	-0.020	0.219	-0.229	-0.018	**0.651**	**0.682**	**1**							
	Phe	0.072	-0.093	0.190	-0.336	-0.099	**0.754**	**0.795**	**0.891**	**1**						
	Thr	-0.102	-0.233	0.146	-0.531	-0.213	**0.790**	**0.702**	**0.762**	**0.827**	**1**					
	Tyr	0.268	0.086	0.324	-0.154	0.117	**0.573**	**0.806**	**0.795**	**0.830**	**0.701**	**1**				
	Val	-0.274	-0.491	-0.191	-0.557	-0.496	**0.850**	**0.626**	**0.628**	**0.724**	**0.615**	**0.481**	**1**			
	Arg	0.406	0.371	0.445	0.157	0.274	0.272	0.422	0.617	0.678	0.446	0.535	0.325	1		
	His	0.604	0.556	0.547	0.477	0.458	0.080	0.313	0.619	0.546	0.266	0.529	0.217	0.793	1	
	Pro	0.337	0.203	0.398	0.004	0.160	0.304	0.389	0.542	0.563	0.581	0.529	0.182	0.517	0.438	1

*The significance threshold based on 249 samples: r = 0.124 at P = 0.05; r = 0.162 at P = 0.01; and r = 0.206 at P = 0.001. P < 0.00001 for those r values bolded.

**Figure 1:**
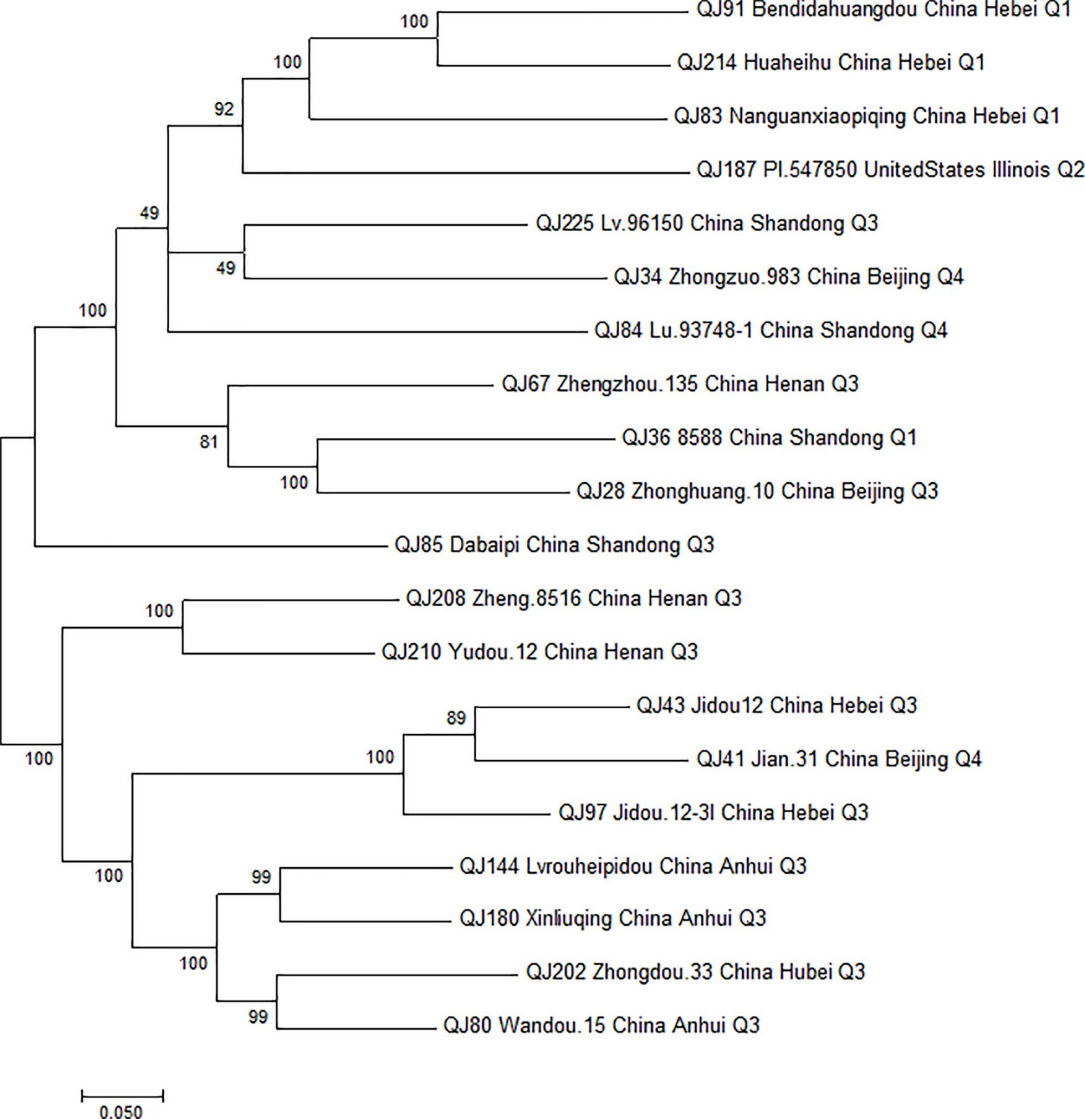
The maximum likelihood tree of the 20 soybean germplasm accessions that ranked in the top three for at least one amino acid concentration among the 249 soybean accessions.

Based on 15 amino acid concentrations, we identified three accessions with the highest concentrations in each of the 15 amino acid concentrations. In addition, we ordered the 249 soybean accessions based on the concentration of each amino acid, and chosen 20 soybean accessions with at least one amino acid concentration topping three among the 249 soybean accessions. These 20 soybean accessions, Zhonghuang 10, Zhongzuo 983, 8588, Jian 31, Jidou 12, Zhengzhou 135, Wandou 15, Nanguanxiaopiqing, Lu 93748-1, Dabaipi, Bendidahuangdou, Jidou 12-3l, Lvrouheipidou, Xinliuqing, PI 547850, Zhongdou 33, Zheng 8516, Yudou 12, Huaheihu, and Lv 96150 would be good amino acid resources for improving amino acids concentration in soybean breeding programs (**Supplementary Table 2** and **Figure 1**).

### Association Mapping and SNP Marker Identification

**Figure 2:**
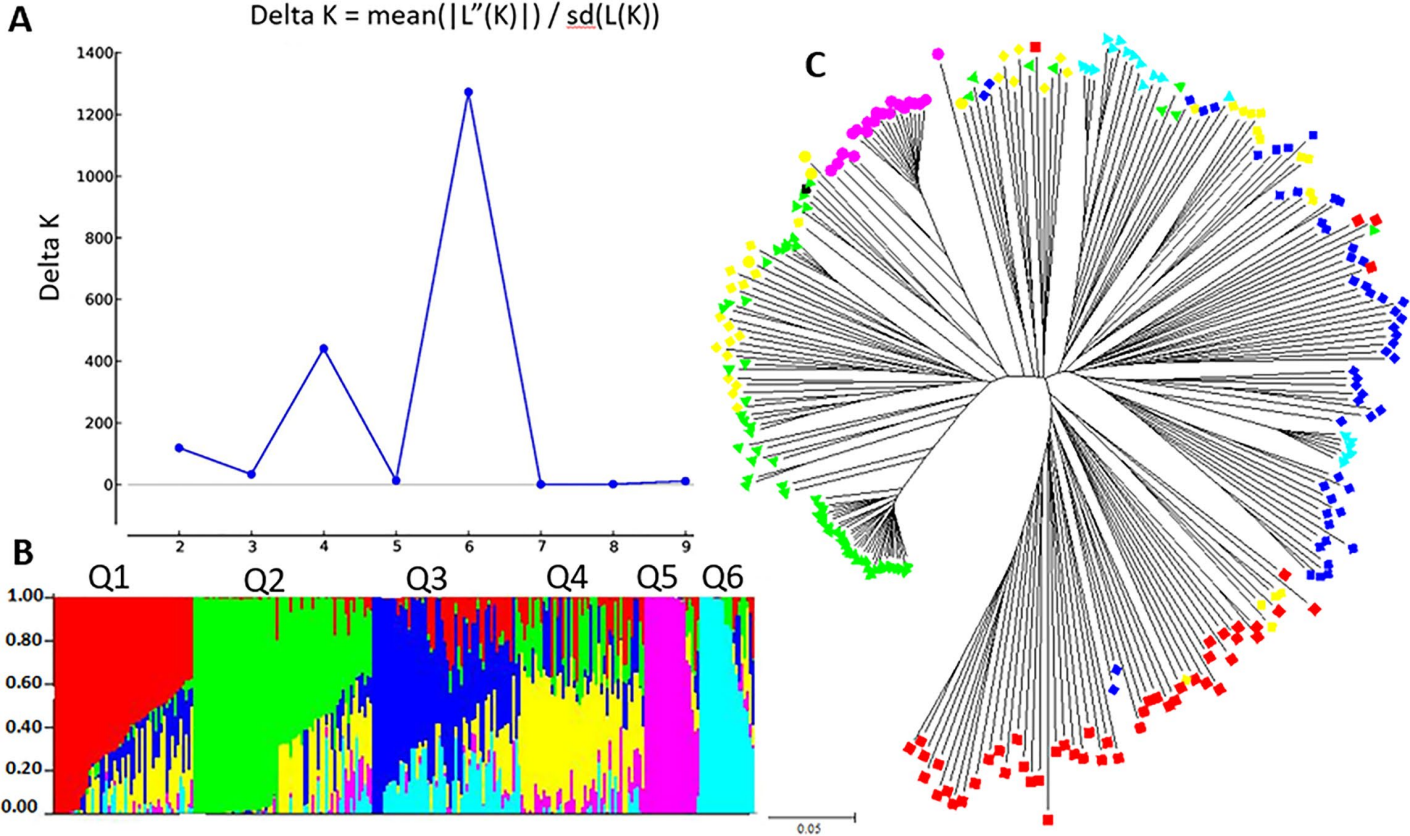
Structure analysis: **(A)** Delta K values for different numbers of populations (K) from the STRUCTURE analysis, x-axes shows different numbers of populations (K), y-axes shows Delta K values for different numbers of populations (K). **(B)** Classification of 249 accessions into six sub-populations using STRUCTURE version 2.3.4, where the x-axis shows accessions, and the y-axis shows the probability (from 0 to 1) of each accession belong to sub-population (Q = K) membership. The membership of each accessions belonging to sub-populations is indicated by different colors (Q1, red; Q2, green; Q3, blue; Q4, yellow; Q5, pink; and Q6: cyan). **(C)** Maximum Likelihood (ML) tree of the 249 accessions drawn in MEGA 6. The color code for each subpopulation is the same as that in the **(B** and **C)**.

The population structure of the 249 soybean accessions was initially inferred using STRUCTURE 2.3.4 (Pritchard et al., 2000) and the peak of delta K was observed at K = 6, indicating the presence of six sub-populations (clusters, Q1-Q6) (**Figure 2A**). In total, 51 of the 249 accessions were assigned to Q1 sub-population with 50 accessions from China; 65 assigned to Q2 with 42 from U.S., 21 from China and two from Korea; 55 assigned to Q3 with 54 cultivars from China; 42 assigned to Q4 with 27 cultivars from China and 12 accessions from U.S.; 21 assigned to Q5 with 16 from U.S.; and 15 to Q6 with all 15 from China (**Figure 2B**, and **Supplementary Table 1**). Phylogenetic analysis of the 249 soybean accessions using MEGA 6 also showed that the clustering of accessions was consistent with that inferred by STRUCTURE (**Figure 2C**).

**Figure 3:**
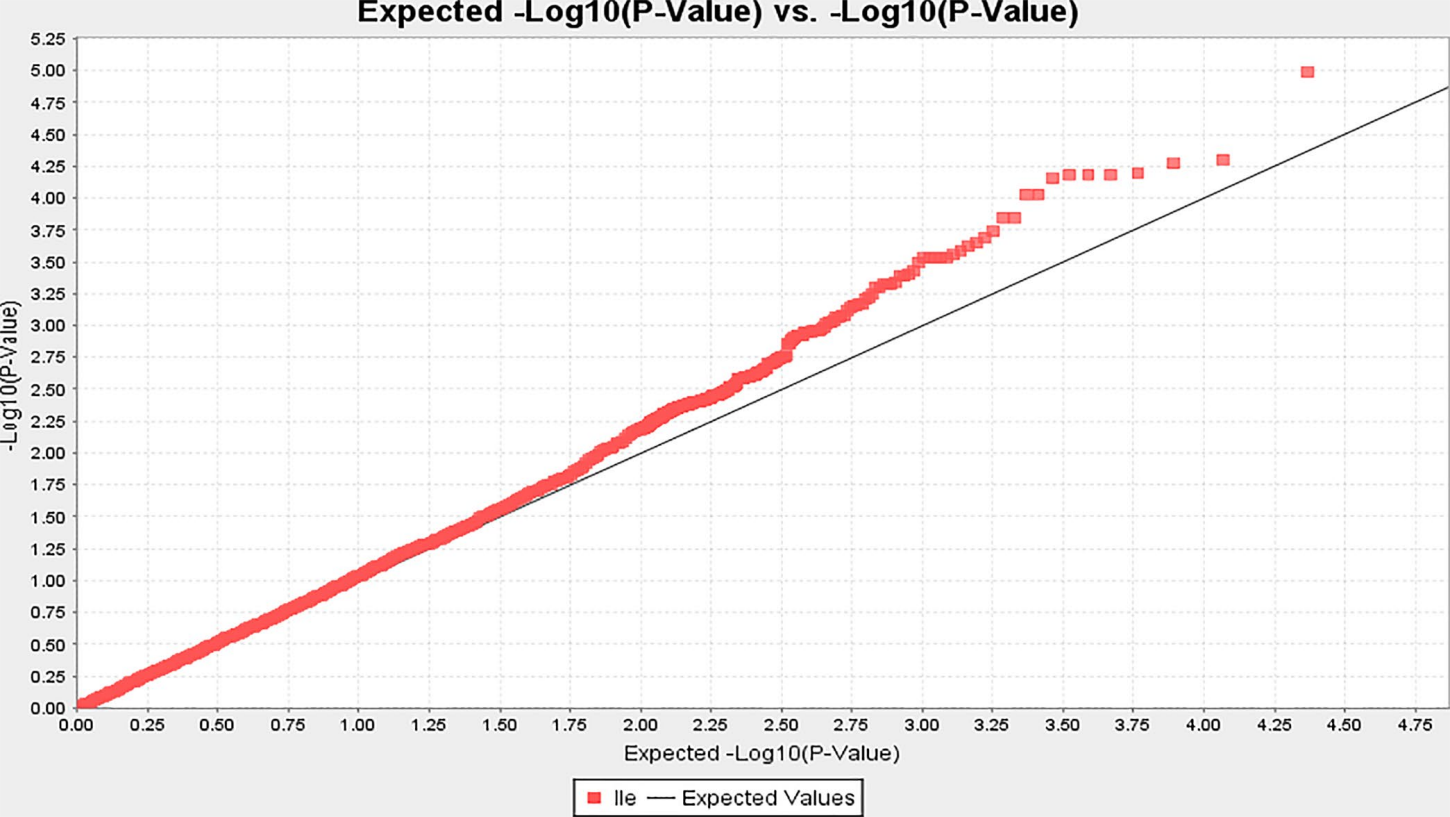
The QQ plot between the expected LOD (-log(P-value)) value and the estimated LOD (log(P-value)) value of amino acid Ile based on 23,279 SNPs as an example (all 15 QQ-plot for the 15 amino acids showed in **Supplementary Figure 3**).

A total of 318 SNP markers consisted of 231 SNPs were associated with the 15 individual amino acid at LOD ≥3 (**Supplementary Table 3** and **Supplementary Figure 2**). Because some SNPs were associated with two or more amino acids as pleiotropic association, the number of SNPs was only 231 (**Table 2**). Of the 318 SNPs, 11 were associated with Ala, 29 with Arg, 9 with Asp, 34 with Glu, 29 with Gly, 19 with His, 51 with Ile (**Figure 3**), 20 with Leu, 14 with Lys, 9 with Phe, 24 with Pro, 11 with Ser, 21 with Thr, 13 with Tyr, and 24 with Val (**Supplementary Table 3** and **Supplementary Figure 3**).

**Table 2:** List of SNP markers associated with each amino acid concentrations at LOD ≥ 3.0, respectively.

SNP ID (chr_pos)	Trait	SNP ID (chr_pos)	Trait	SNP ID (chr_pos)		SNP ID (chr_pos)	Trait
Gm01_33262451	Ile	Gm07_4574178	Ser	Gm13_39628016	Ile	Gm16_6737312	Thr
Gm01_45320366	Ile	Gm07_5923593	Arg	Gm13_39628019	Ile	Gm16_780258	Ile, Val
Gm01_53597652	Gly	Gm08_14156183	Ala, Glu	Gm13_39628049	Ile	Gm17_14444779	Pro
Gm01_571041	His	Gm08_1969577	Glu	Gm13_39628054	Ile	Gm17_23967094	Tyr
Gm01_571048	His	Gm08_3446621	Lys	Gm13_40242572	Pro	Gm17_2459036	Pro
Gm02_15368490	Val	Gm08_43340095	Lys	Gm13_40242573	Pro	Gm17_2475262	Pro
Gm02_45763574	His	Gm08_45648867	Asp	Gm13_40242709	Pro	Gm17_2674908	Pro
Gm02_47034495	Ile, Thr, Val	Gm08_8091680	Glu	Gm13_41203949	His, Lys, Pro	Gm17_37708047	His, Pro
Gm02_48215047	Pro	Gm08_8480396	Gly	Gm13_7762318	Arg	Gm17_37708072	His, Pro
Gm02_49856130	Ile, Val	Gm08_8538031	Gly	Gm14_28719225	Gly	Gm17_37708077	His, Pro
Gm02_50224425	Arg	Gm09_43473530	Ala	Gm14_42728555	Glu	Gm17_37708117	His, Pro
Gm02_50269310	Arg	Gm09_43488824	Ala, Asp	Gm14_42900467	Glu	Gm17_37712338	Arg, Pro
Gm02_5190606	Ile	Gm10_12029489	Ala	Gm14_43163207	Glu	Gm18_1231280	Ile
Gm02_6671113	Gly	Gm10_35214322	Val	Gm14_43163233	Glu	Gm18_12797087	Thr
Gm02_6721375	Asp, Gly, Ile, Ser	Gm10_44070578	Ile	Gm14_43163234	Glu	Gm18_1449038	Glu, Ser
Gm03_36272238	Thr	Gm10_45237186	Ile	Gm14_43163255	Glu	Gm18_14877256	Phe
Gm03_36417795	Thr	Gm10_46037693	Glu	Gm14_43163263	Glu	Gm18_1564092	Glu
Gm03_36465287	Thr	Gm10_46037954	Ala, Glu	Gm14_43163268	Glu	Gm18_2026494	Thr
Gm03_36530224	Pro	Gm10_46045322	Glu	Gm14_43163302	Glu	Gm18_23446982	Ile
Gm03_40600088	Pro	Gm10_47770916	Arg	Gm14_43163309	Glu	Gm18_23680823	Ile
Gm03_40600203	Pro	Gm10_48103776	Asp, Glu, His, Ser	Gm14_43163317	Glu	Gm18_45637951	Ile
Gm03_6537448	Arg, His	Gm10_48367427	Ser	Gm14_670550	Arg	Gm18_54941806	Leu
Gm04_29795804	Gly, Ile, Thr	Gm10_4877563	Arg	Gm14_670770	Arg	Gm18_54941806	Tyr
Gm04_3722529	Pro	Gm10_4877661	Arg	Gm15_42452169	Lys, Phe, Val, Lys, Phe, Val	Gm18_55570016	Arg
Gm04_43205897	Gly	Gm10_50892012	Glu	Gm15_46888773	His	Gm18_57994827	Arg
Gm04_43205900	Gly	Gm10_50945017	Glu	Gm15_6364620	Gly	Gm18_57994865	Arg
Gm04_43207187	Gly, Ile, Thr, Phe	Gm10_50945124	Glu	Gm15_6364624	Gly	Gm18_58356668	Gly
Gm04_43207248	Gly, Ile, Phe, Thr	Gm10_6088950	Arg	Gm15_6364658	Gly	Gm18_61819070	Leu
Gm04_43247307	Gly	Gm10_6127825	Arg	Gm15_6364660	Gly	Gm18_61846089	Leu
Gm04_43247365	Gly	Gm10_6158335	Arg	Gm15_6364671	Gly	Gm18_61846097	Leu
Gm04_45172948	Ile	Gm11_17324386	Leu	Gm16_19302037	Ile	Gm18_61846199	Leu
Gm05_1131617	Thr, Tyr, Leu	Gm11_36252840	Lys, Phe, Tyr	Gm16_19309923	Ile, Val	Gm18_61846240	Leu
Gm05_1364762	Gly	Gm11_36391557	Pro	Gm16_19310296	Ile, Val	Gm18_61846255	Leu
Gm05_1956615	Glu, Ser	Gm11_38372080	Ile	Gm16_19474288	Ile	Gm18_61846357	Leu
Gm05_21977894	Ile, Val	Gm12_1283279	Ile	Gm16_26668643	Pro	Gm18_829983	Leu
Gm05_36368612	Tyr	Gm12_1966701	Leu, Val	Gm16_26668804	Pro	Gm18_849773	Leu
Gm06_14669414	Lys	Gm12_2246393	Ile, Leu, Phe, Thr, Val	Gm16_26760058	Pro	Gm18_8944865	Lys, Thr
Gm06_1655912	Arg, Tyr	Gm12_2246402	Thr	Gm16_27656811	Ile, Leu	Gm19_14283927	Ile, Val
Gm06_20941559	Glu	Gm12_2246405	Thr	Gm16_27675722	Ile, Leu	Gm19_34599708	Ala
Gm06_289575	Gly	Gm12_2246408	Thr	Gm16_28109123	Ile	Gm19_35491961	Ile, Val
Gm06_399885	Ala, Asp, Gly	Gm12_2246409	Thr	Gm16_30033799	Val	Gm19_35491974	Ile, Val
Gm06_46691924	Gly	Gm12_37250318	Ile, Leu	Gm16_31565242	Lys, Val	Gm19_35491994	Ile, Val
Gm06_48160139	His	Gm12_37253606	Ile	Gm16_31565425	Lys, Val	Gm19_35491998	Ile, Val
Gm06_48405502	Gly	Gm12_37699937	Pro	Gm16_32344691	Arg	Gm19_35492018	Ile, Val
Gm06_49021688	Gly	Gm12_37700016	Pro	Gm16_32636611	Arg	Gm19_35492028	Ile, Val
Gm06_582930	Gly	Gm12_4525326	Arg	Gm16_32891444	Arg	Gm19_35492061	Ile, Val
Gm07_16345870	Phe	Gm12_4525341	Arg	Gm16_33487136	Arg, His	Gm19_35492063	Ile, Lys, Val
Gm07_3374472	Gly	Gm12_9802063	Ile	Gm16_33595082	Arg, His	Gm19_36853376	Ala, Ser
Gm07_3374492	Gly	Gm13_17646967	Asp, Ile, Ser	Gm16_33670373	Asp	Gm19_36856526	Ala, Glu, Ser
Gm07_36388230	Asp	Gm13_21744787	Asp, Glu, Ser	Gm16_33761779	Arg, His	Gm19_38354186	Tyr
Gm07_36390103	Glu	Gm13_21758530	Ala, Asp	Gm16_33853366	Arg, HIs	Gm19_41048945	Glu
Gm07_36524487	Glu	Gm13_22508206	Arg	Gm16_35244130	His	Gm20_31240721	Leu, Tyr
Gm07_36542987	Glu	Gm13_38830655	Lys	Gm16_35747794	Ser	Gm20_31240801	Tyr
Gm07_36543902	Glu	Gm13_39627980	Ile	Gm16_36927834	Tyr	Gm20_31387086	His
Gm07_36633143	Glu	Gm13_39627983	Ile	Gm16_36927871	Tyr	Gm20_35630363	Leu
Gm07_36633260	Glu	Gm13_39627986	Ile	Gm16_6737154	His, Lys, Thr	Gm20_42531505	Gly, Ile, Leu, Tyr, Phe, Thr
Gm07_3811476	Arg	Gm13_39628010	Ile	Gm16_6737218	Thr	Gm20_42569717	Lys, Tyr
Gm07_39077446	Ala	Gm13_39628014	Ile	Gm16_6737289	Thr		

The total number of haplotype blocks was 3,458 based on 23,279 SNPs, the 231 SNPs were positioned in 85 of these haplotype blocks (**Supplementary Table 3**). Many haplotype blocks contained more than two SNP markers. For example, Gm12_4525341 and Gm12_4525326 were in the same haplotype block and associated with Arg; Gm06_289575, Gm06_399885, and Gm06_582930 were in the same haplotype block on Chr 6 and were associated with Gly (**Supplementary Table 3**).

The number of the haplotype blocks varied among chromosomes, e.g. 12 of the 85 haplotype blocks were on Chr 16; 11 haplotype blocks on Chr 18; 1 on Chrs 6 and 9. Twenty of the 85 haplotype blocks had significant association with more than one amino acids, e.g. Gm20_42531505 on the Chr. 20_Block 2 was significantly associated with Thr, Gly, Ile, Tyr, Leu, Phe; Two SNP markers, Gm04_43207248 and Gm04_43207187 in the Chr. 4_Block 3, were significantly associated with Ile, Phe, Gly and Thr; and two markers, Gm15_42452169 and Gm15_42452285 in the Chr. 15_Block 2 associated with Val, Phe and Lys (**Supplementary Table 4**).

Based on phenotypic patterns of the amino acid concentration among accessions, the 15 amino acids could be divided into two groups which were showed in phenotypic variance section. SNP markers associated with amino acids in each group were also found. Twenty-five SNP markers were associated with five amino acids, Ala, Asp, Glu, Gly, and Ser in group one (**Table 3**), and 28 SNP markers with seven amino acids, Ile, Leu, Lys, Phe, Thr, Tyr, and Val in group two (**Table 4**). The SNP markers in each group can be used to simultaneously select multiple amino acids within the group. Such as Gm10_48103776 was associated with five amino acids, Ala, Asp, Glu, Gly, and Ser in group one with LOD values of 2.93, 3.15, 3.51, 2.35, and 3.60, respectively (**Table 3**) and it can be used to simultaneously select soybean lines with higher contents of the five amino acids in soybean breeding progress. For group two, such as Gm20_42531505 was associated with seven amino acids, Ile, Leu, Lys, Phe, Thr, Tye, and Val with LOD values of 3.53, 4.55, 2.89, 4.79, 5.04, 3.87, and 2.10, respectively (**Table 4**), indicating that it can be used to simultaneously select the soybean lines with higher contents of seven amino acids. Meanwhile, both phenotypic and genetic data supported there were two groups of amino acids existed in soybean.

**Table 3:** Twenty-five SNP markers associated with five amino acids of group one, simultaneously.

SNP ID	Chr	Position (bp)	Ala	Asp	Glu	Gly	Ser
Gm02_6671113	2	6671113	2.19*	2.83	0.68	3.46	2.48
Gm02_6721375	2	6721375	2.85	5.41	0.83	3.36	4.63
Gm06_399885	6	399885	3.14	2.58	1.86	4.15	2.35
Gm07_36388230	7	36388230	2.38	3.06	2.13	2.29	2.95
Gm07_36542987	7	36542987	1.18	2.36	3.68	2.20	2.01
Gm07_36633143	7	36633143	2.06	2.13	3.26	0.83	2.15
Gm07_36633260	7	36633260	2.06	2.13	3.26	0.83	2.15
Gm10_46037693	10	46037693	2.88	2.10	3.84	2.10	2.35
Gm10_46037954	10	46037954	3.07	2.38	3.86	2.70	2.44
Gm10_48103776	10	48103776	2.93	3.15	3.51	2.35	3.60
Gm13_21744787	13	21744787	2.31	3.05	3.03	2.66	3.27
Gm13_21758530	13	21758530	3.12	3.18	2.92	2.04	2.97
Gm14_43163207	14	43163207	2.10	2.46	3.83	0.90	2.17
Gm14_43163233	14	43163233	2.10	2.46	3.83	0.90	2.17
Gm14_43163234	14	43163234	2.58	2.54	4.37	1.33	2.57
Gm14_43163255	14	43163255	2.58	2.54	4.37	1.33	2.57
Gm14_43163263	14	43163263	2.10	2.46	3.83	0.90	2.17
Gm14_43163268	14	43163268	2.58	2.54	4.37	1.33	2.57
Gm14_43163302	14	43163302	2.10	2.46	3.83	0.90	2.17
Gm14_43163309	14	43163309	2.10	2.46	3.83	0.90	2.17
Gm14_43163317	14	43163317	2.58	2.54	4.37	1.33	2.57
Gm16_35747794	16	35747794	2.78	2.22	1.46	2.40	3.02
Gm18_1449038	18	1449038	2.34	2.93	3.22	2.74	3.39
Gm19_36853376	19	36853376	3.07	2.37	2.51	1.17	3.52
Gm19_36856526	19	36856526	3.04	2.42	3.64	1.97	3.54

*LOD (-log(P-value)) from MLM of Tassel.

**Table 4:** Twenty-eight SNP markers associated with seven amino acids of group two, simultaneously.

SNP ID	Chr.	Position (bp)	Ile	Leu	Lys	Phe	Thr	Tyr	Val
Gm03_36417795	3	36417795	1.95*	1.34	2.06	2.91	3.38	2.44	2.44
Gm03_36465287	3	36465287	2.98	1.95	2.46	2.85	3.68	2.68	2.34
Gm04_43207187	4	43207187	3.39	1.95	2.52	3.57	3.17	2.46	2.39
Gm04_43207248	4	43207248	3.39	1.95	2.52	3.57	3.17	2.46	2.39
Gm04_45172948	4	45172948	3.11	2.29	2.11	2.15	2.06	1.42	1.95
Gm05_1131617	5	1131617	2.74	3.69	2.54	2.51	3.32	3.24	1.15
Gm05_1364762	5	1364762	2.70	1.78	2.52	2.13	2.78	2.46	1.72
Gm05_21977894	5	21977894	3.59	1.72	2.67	2.18	2.64	2.60	3.28
Gm08_3446621	8	3446621	2.39	0.90	3.99	2.73	2.05	1.30	2.66
Gm11_36252840	11	36252840	1.13	2.48	3.94	3.48	2.62	4.30	1.26
Gm12_1966701	12	1966701	2.95	3.24	1.70	2.20	1.51	2.38	3.13
Gm12_2246393	12	2246393	3.40	3.02	2.36	3.27	4.13	2.64	3.39
Gm12_9802063	12	9802063	3.32	2.04	1.81	2.38	2.64	1.38	2.12
Gm15_42452169	15	42452169	2.19	1.55	3.80	3.35	2.06	1.87	3.79
Gm16_6737218	16	6737218	2.58	1.11	2.89	2.24	3.59	1.90	2.19
Gm16_27675722	16	27675722	3.03	3.63	1.62	2.39	2.30	2.12	1.35
Gm18_1231280	18	1231280	3.17	0.91	2.15	2.81	2.35	1.54	2.34
Gm18_14877256	18	14877256	2.86	2.18	2.67	3.09	2.27	1.92	1.60
Gm18_54941806	18	54941806	2.45	3.91	2.47	2.54	2.27	3.60	1.52
Gm19_35491974	19	35491974	4.20	0.59	2.48	2.38	2.90	0.95	4.17
Gm19_35491994	19	35491994	4.03	0.64	2.51	2.26	2.61	0.93	3.99
Gm19_35491998	19	35491998	4.19	0.62	2.51	2.35	2.82	0.88	4.04
Gm19_35492018	19	35492018	4.19	0.62	2.51	2.35	2.82	0.88	4.04
Gm19_35492028	19	35492028	4.19	0.62	2.51	2.35	2.82	0.88	4.04
Gm19_35492061	19	35492061	4.30	0.64	2.71	2.50	2.94	0.97	4.23
Gm19_35492063	19	35492063	4.15	0.68	3.22	2.81	2.98	1.32	3.35
Gm20_42531505	20	42531505	3.53	4.55	2.89	4.79	5.04	3.87	2.10
Gm20_42569717	20	4256971	1.64	2.09	3.43	2.92	2.47	3.00	1.42

*LOD (-LOG(P-value)).

### Candidate Gene Selection

The linkage disequilibrium (LD) of soybean genome was analyzed, the average distance of markers at half of the maximum LD decay rate was about 200kb. Considering the LD decay value may vary from genomic region to region, we used the 10kb windows as previously reported (Xie et al., 2018). We identified 704 genes with all or partial sequence within the 10 kb windows that flanked each of the 217 out of 231 unique SNPs associated with one or more amino acids (**Supplementary Table 5**) and the other 14 SNPs did not have any candidate genes at the 10 kb windows on the chromosomes.

Based on gene annotations of the soybean whole genome assembly Gmax_275_Wm82.a2.v1 from Soybase and PMN (Phytozome v12: Gmax_275_Wm82.a2.v1.protein.fa), we found that 15 SNPs were in 14 genes related to amino acid metabolism in gene ontology annotation terms (**Supplementary Table 6**), e.g. in the region flanking the SNP Gm03_36417795, there was a candidate gene “Glyma03g28476 (Glyma 1.1)/Glyma.03g129100 (Glyma 2.0)” encoding for pyrroline-5-carboxylate reductase (Delauney and Verma, 1990)^13^ (**Supplementary Table 6**). This enzyme catalyzes the last step of L-proline biosynthesis through the L-glutamate degradation pathway. In the region flanking the SNP Gm03_36465287, there was a gene Glyma03g28530 (Glyma 1.1)/Glyma.03g129700 (Glyma 2.0) encoding β L-selenocystathionase, a key enzyme catalyzing L-homocysteine and L-cysteine interconversion. L-homocysteine and L-cysteine interconversion is an intermediate step for conversion between methionine and cysteine (McCluskey et al., 1986)^14^ (**Supplementary Table 6**).

### Genomic Selection for Amino Acid Concentration Based on RR-BLUP in rrBLUP

Based on RR-BLUP in rrBLUP, the GEBV of each amino acid was estimated using three different sets of SNPs, i.e. 23,279 SNPs, 231 SNP markers associated with 15 amino acid, and SNP markers associated with an individual amino acid.

The correlation coefficients between GEBV and observed value varied among amino acids based on all 23,279 SNPs (column-2 in **Table 5**), the r value was 0.61 for Arg; 0.50 for Phe; between 0.35 and 0.50 for His, Lys, Thr and Tyr; between 0.25 and 0.35 for Ala, Glu, Ile, Leu, Pro, and Val; and less than 0.25 for Asp, Gly, and Ser. The r values for most amino acids were less than 0.5, suggesting GS prediction accuracy for most amino acids was low based on genome-wide random SNPs.

**Table 5:** The averaged correlation coefficient (r) among 15 amino acids between the observed values (each amino acid content) and the GEBVs predicted from (1) all 23,279 SNPs, (2) the 231 SNP markers, and (3) only the associated SNP markers with the specific amino acid content using RR-BLUP in rrBLUP software, and from (4) the 231 SNP markers in reference set (training set) and inference set (validation set) using CBLUPin Gapit.

	RR-BLUP in rrBLUP			CBLUP in Gapit	
	23279 SNPs in 249 accessions	231 SNPs associated with amino acids	Associated SNPs*	231 SNPs in training set	231 SNPs in validation set
Ala	0.30	0.45	0.33	0.76	0.52
Arg	0.61	0.68	0.61	0.80	0.59
Asp	0.22	0.53	0.41	0.77	0.57
Glu	0.31	0.48	0.48	0.74	0.52
Gly	0.23	0.56	0.35	0.79	0.60
His	0.46	0.57	0.46	0.76	0.55
Ile	0.25	0.61	0.53	0.80	0.61
Leu	0.30	0.53	0.49	0.77	0.53
Lys	0.42	0.61	0.54	0.82	0.59
Phe	0.50	0.68	0.35	0.84	0.68
Pro	0.26	0.49	0.34	0.76	0.46
Ser	0.18	0.47	0.42	0.73	0.51
Thr	0.39	0.63	0.50	0.85	0.63
Tyr	0.36	0.54	0.44	0.81	0.57
Val	0.25	0.52	0.53	0.75	0.54
Average	0.34	0.56	0.45	0.78	0.56

*Associated SNPs signifies that the average correlation coefficient (r) for each amino acid in column-4 was calculated with the SNP markers only associated with the individual amino acid to predict the GEBV for each amino acid, such as for r = 0.33 for Ala, which was calculated from 11 associated SNPs.

**Figure 4:**
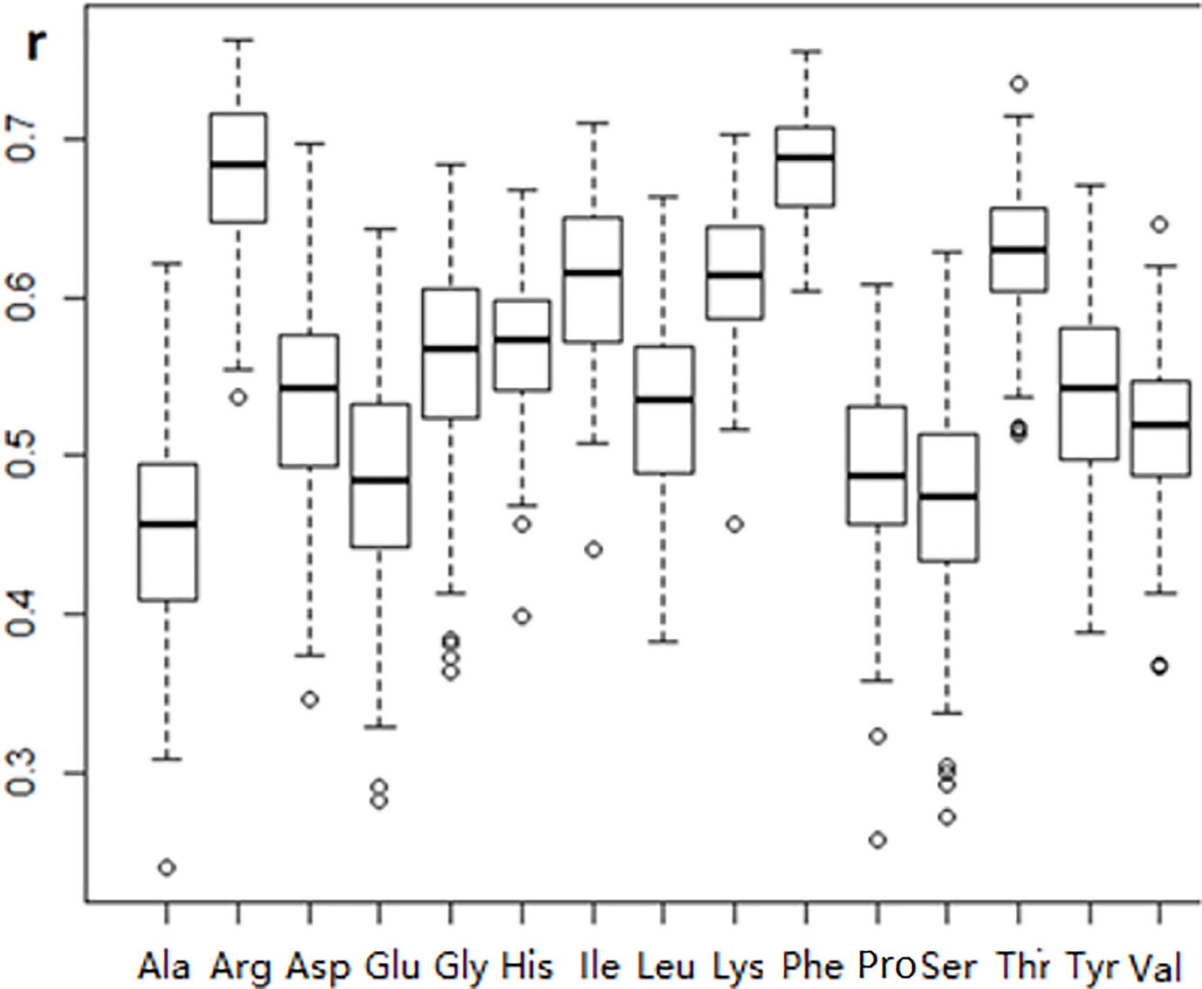
The correlation coefficient (r) among 15 amino acids between the observed values (each amino acid concentration) and the GEBVs predicted from the 231 SNP markers using RR-BLUP in rrBLUP software.

The correlation coefficients between GEBV and observed value of the 15 amino acids were equal or higher from 231 SNPs than those from the 23,279 SNPs (column-3 vs column-2 in **Table 5**). The *r* value was larger than 0.6 for Arg, Ile, Lys, Phe, and Thr, and between 0.5 and 0.6 for Asp, Gly, His, Leu, Tyr, and Val, indicating that associated markers were more efficient to predict amino acids for soybean lines than all the SNPs (**Figure 4** and column-3 in **Table 5**).

Of the 231 SNPs, a total of 171, 42, 12, 4, 1 and 1 SNPs were associated with only one, two, three, four, five, and six amino acids, respectively. A total of 11, 29, 9, 34, 29, 19, 51, 20, 14, 9, 24, 11, 21, 13, and 24 SNP markers were associated with Ala, Arg, Asp, Glu, Gly, His, Ile, Leu, Lys, Phe, Pro, Ser, Thr, Tyr, and Val, respectively (**Supplementary Table 3**). We used the SNP markers only associated with individual amino acid to predict the GEBV for each amino acid, the r values for the 14 amino acids were higher than those from the 23,279 SNPs except for Phe, but equal or lower than those from the 231 SNP markers except for Val (**Table 5**).

T-test was conducted to compare the r values from the 231 SNPs and from the all 23,279 SNPs and found that the r value from the 231 SNPs in column-3 for each amino acids was significantly higher than that in column-2 from all SNPs with P = 0.01 level in **Table 5**, indicating that using the associated SNPs had better prediction for GS than using all randomly SNPs (**Table 5**).

### Genomic Selection for Amino Acid Concentration Based on CMLM in GAPIT

**Figure 5:**
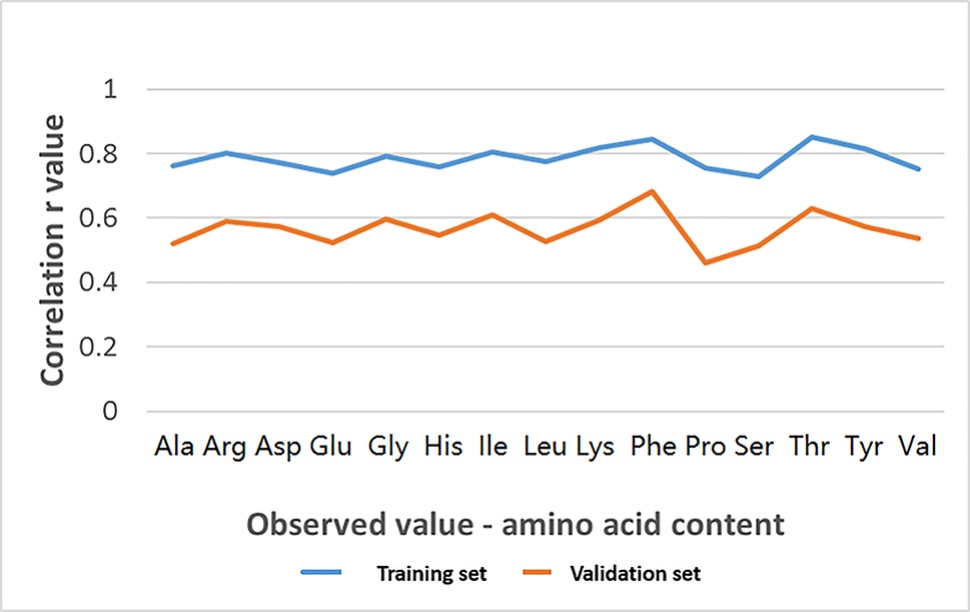
The average correlation coefficient (r) among 15 amino acids between the observed values (each amino acid concentration) and the GEBVs predicted in both training set and validation set from the 231 SNP markers using cBLUP method in GAPIT.

Based on cBLUP method using CMLM in GAPIT, the average r was estimated (**Table 5** and **Figure 5**). The average correlation coefficient in the training set was greater than 0.7 and was higher than those in validation set. The average values in validation set were greater than 0.5 for amino acids except for Pro.

Two comparisons were tested to validate the stability of GS using different estimate methods and approaches: (1) RR-BLUP in rrBLUP vs cBLUP in Gapit, and (2) self-validation (training set by itself) vs cross-validation (training set). For the first comparison, the 15 r values in column-3 (“231 SNPs in 249 accessions”) was compared to those in column-6 (“231 SNPs in validation set”) in **Table 5** and we found a strong association between the average r values from RR-BLUP in rrBLUP and from cBLUP in Gapit (r = 0.85) based on the 231 associated SNPs. For the second comparison, the 15 r values in column-5 (“231 SNPs in training set”) was compared to those in column-6 (“231 SNPs in validation set”) in **Table 5** and we found a strong association between the average r values from cBLUP in Gapit (r = 0.84) based on the 231 associated SNPs. The strong association with high r value >0.8 between different methods and approach showed that we can use the 231 SNPs to select high amino acid content in soybean through GS.

## Discussion

### Application of Marker-Assisted Selection to Genetic Improvement of Soybean

Previous studies using bi-parental segregating populations have identified QTLs controlling 15 amino acids in soybean seeds (Panthee et al., 2006; Fallen et al., 2013; Khandaker et al., 2015; Warrington et al., 2015). The QTL were associated with 84 molecular markers on 14 chromosomes (**Supplementary Table 7**). In this study, we identified 231 unique SNP markers significantly associated with 15 amino acids (**Supplementary Table 3**). Eight SNPs were in the same regions of SSR markers that were associated with amino acid concentrations reported by Panthee et al. (2006), e.g. the SNP marker, Gm07_4574178 (located at 4.5 Mb on chr 7) associated with Ser was near the SSR marker, Satt 567 (located at 63,663 bp on chr 7), Gm19_41048945 at 41 Mb on chr 19 for Glu was near Satt076 at 374,148 bp of chr 19; Gm02_15368490 at 15,368,490 bp on chr 2 for Val near Satt537; Gm01_45320366 at 45,320,366 bp on chr 1 for Ile near Satt203; Gm19_35491961 at 35,491,961 bp on chr 19 for Ile near Satt313; Gm02_50269310 at 50,269,310 bp on chr 2 for Arg also near Satt274 and Satt196; and Gm09_43488824 at 43,488,824 bp on chr 9 for Asp near Satt196 (Panthee et al., 2006). Two SNP markers, Gm09_43488824 at 43,488,824 bp on chr 9 for Asp and Gm10_48103776 at 48,103,776 bp on chr 10 for His were close to the regions controlling the two amino acids reported by Fallen et al. (2013) (**Supplementary Table 7**). In addition, Gm09_43488824 at 43,488,824 bp on chr 9 associated with Asp was in the regions reported by Panthee et al. (2006) and Fallen et al. (2013).

As GWAS for amino acid concentrations in soybean, Zhang et al. (2018) reported that 54 SNPs, as 92 markers were associated with 18 amino acids; 38 of the 54 SNPs associated with only one amino acid; and 11 SNPs associated with 2 to 12 amino acids. The SNP markers for each amino acid were located at one chromosome such as Pro or Ser, nine chromosomes such as Arg or Asp, up to 11 chromosomes such as Try (**Supplementary Table 7**). Comparisons with the SNP markers associated with amino acids reported by Zhang et al. (2018), most of SNP markers were located at different regions of soybean chromosomes. However, there were four regions similar to our results: (1) 3.71–3.82 Mb of chr 7 for Arg; (2) 33.85–35.73 Mb of chr 16 for Arg; (3) 16.28–17.65 Mb of chr13 for Asp; and (4) 8.27–9.33 Mb of Chr 8 for Gly. From our study, the SNP marker Gm07_3811476 was associated with Arg at 3,811,476 bp on chr 7, which was near with around 98 kb to the SNP markers ss715597475 at 3,713,267 bp on chr7 for Arg reported by Zhang et al. (2018). Another SNP, Gm16_33853366 close to ss715624781 with 1.87 M distance on chr 16 was also associated with Arg; Gm16_33853366 was at 33,853,366 bp and ss715624781 at 35,721,993 bp on chr 16. For Asp, the Gm13_17646967 at 17,646,967 bp was close to ss715616790 at 16,286,313 bp with a distance 1,360,654 bp on chr 13. The SNP markers, Gm08_8480396 and Gm08_8538031 associated with Gly from this study were close to the two SNP markers, ss715602750 and ss715602851 with Gly (Zhang et al., 2018) and the four markers are located at a region with one Mb distance on chr 8. Thus, the four regions were validated to be associated with one of the amino acid, Arg, Asp, or Gly.

These SNPs identified for 15 amino acids in this study can be used as molecular markers to select lines with high amino acids content through marker-assisted selection (MAS). PCR-based KASP SNP genotyping can be used in soybean breeding program to select high amino acids through MAS. Targeted region sequencing such as tGBS (targeted genotyping-by-sequencing) (Simko et al., 2018) can also be used for MAS and GS based on the sequences flanking these SNPs (Ott et al., 2017).

From this study, 14 candidate genes were found to be related to amino acid metabolism based on gene annotations from Soybase and PMN with gene ontology annotation terms using the DNA sequences in the 15 regions with the 15 SNPs in column-B of **Supplementary Table 7** significantly associated with amino acids (**Supplementary Table 6**). Our further research will develop the molecular markers such as PCR-based assays or targeted region sequencing to validate these candidate genes in our association panel and others. Gene-silence through CRISPR/Cas9 may be used as an approach to validate these candidate genes.

### Genomic Selection

Prediction accuracy is the main parameter to measure the performance of GS (Jarquin et al., 2016; Zhang et al., 2016; Duhnen et al., 2017). Prediction accuracy is affected by several factors including GS models, marker density, level of LD, QTL number, the population size specially the training population size, relationship between training population and validation population, and trait heritability (Jarquin et al., 2016).

Zhang et al. (2016) estimated prediction accuracy (r value) of seed size based on 309 soybean accessions and reported r = 0.85 when 2000 SNPs or 31,045 SNPs were included, r = 0.8 when 1000 SNPs or 500 SNPs were used. They also identified 48 SNPs on 12 chromosomes associated with seed size based on GWAS. The r value ranged from 0.64 to 0.74 when 5, 10, and 15 of the 48 SNP markers were used, which were 25% higher than those calculated from the same number of randomly selected SNPs. Our results showed that the highest r value (0.56) was obtained based on the model including 231 SNPs signific antly associated with one or multiple amino acids, followed by the model including SNPs significantly associated with individual amino acid (r = 0.45), and the least was the model including all SNPs (r = 0.34). A t-test showed r values were significantly different among the sets.

We also estimated the GEBV and r values using the cBLUP in GAPIT. Based on the set of 231 SNPs, the correlation coefficient was greater than 0.7 in the training population and greater than 0.5 in validation population. The high correlation between the reference and inference (0.84) based on 15 amino acids, further confirmed the reliability of the GS. A high correlation (0.85) of the prediction accuracy between rrBLUP and GAPIT based on 231 SNPs, indicated that both RR-BLUP in rrBLUP or cBLUP in GAPIT were consistent.

## Conclusion

In this study, soybean accessions with high concentrations of amino acids in seeds, and molecular markers associated with individual and groups of amino acids were identified. These soybean accessions with high amino acid concentrations could be used as parents in soybean breeding programs. The SNP markers strongly associated with the concentrations of the amino acids could be used to improve the nutritional quality of soybean through marker-assisted selection. In addition, fourteen candidate genes that were related to amino acid metabolism were also identified. These candidate genes will lead to a better understanding of the molecular mechanisms that control amino acids metabolism in soybean seeds. Genomic selection analysis of amino acid concentration showed that the selection efficiency of amino acids based on the markers significantly associated with 15 amino acids was higher than that based on genome-wide random markers or markers only associated with an individual amino acid. These results suggest that including a set of markers significantly associated with multiple amino acids in genomic selection is likely to help breeders to efficiently select soybean varieties with improved amino acid content.

## Data Availability Statement

SNP data can be found in the ENA using accession number PRJEB34546 (www.ebi.ac.uk/ena/data/view/PRJEB34546).

## Ethics Statement

All data and materials are not related to human and animals. This research is not related to any plant specimens to be deposited as vouchers or any other association for this section.

## Author Contributions

JQ, AS, YC, FW, and WR carried out phenotyping and genotyping. AS, JQ, SL, and QS analyzed the data. JQ composed the draft of the manuscript. MZ and CY directed and managed this research. AS and QJS reviewed and edited the manuscript. All authors have read, made corrections, and approved the final manuscript.

## Funding

The authors would like to thank Prof. Lijuan Qiu (Chinese Academy of Agricultural Sciences) for providing seeds of 249 soybean accessions. This study was supported by the Hebei Province Natural Science Foundation for Distinguished Young Scholars (C2014301035), National Natural Science Foundation of China (31100880), and Key Project of the Natural Science Foundation of Hebei Province (C2012301020).

## Conflict of Interest

The authors declare that the research was conducted in the absence of any commercial or financial relationships that could be construed as a potential conflict of interest.

## Footnotes

http://www.soystats.com, accessed on August 10, 2019

https://www.fediol.eu, accessed on August 10, 2019

https://www.soybase.org/GlycineBlastPages/archives/Gma1.01.20140304.fasta.zip

https://www.soybase.org/GlycineBlastPages/index.php?db_select=Gma1.01

https://www.jmp.com/en_us/software/genomics-data-analysis-software.html; accessed on August 10, 2019

https://web.stanford.edu/group/pritchardlab/structure_software/release_versions/v2.3.4/html/structure.html, accessed on August 10, 2019

http://taylor0.biology.ucla.edu/structureHarvester/

http://www.maizegenetics.net/tassel

https://github.com/rossibarra/r_buffet/blob/master/LDit.r, verified on May 10, 2018

https://www.soybase.org/dlpages/; accessed on August 10, 2019

https://www.plantcyc.org/; accessed on August 10, 2019

https://www.soybase.org/correspondence/index.php12, accessed on August 10, 2019

https://link.springer.com/article/10.1007/BF00259392, accessed on August 10, 2019

https://doi.org/10.1016/0031-9422(86)80067-X, accessed on August 10, 2019

## Abbreviations

SNP Single nucleotide polymorphism; GWAS Genome-wide association study; AA Amino acids; Ala Alanine; Arg Arginine; Asp Aspartic acid; Glu Glutamic acid; Gly Glycine; His Histidine; Ile Isoleucine; Leu Leucine; Lys Lysine; Phe Phenylalanine; Pro Proline; Ser Serine; Thr Threonine; Tyr Tyrosine; Val Valine; SSR Simple sequence repeat; MAS Marker-assisted selection.
