# Supplementary material for: Genome Wide Association Study and Genomic Selection of Amino Acid Concentrations in Soybean Seeds
Source: Front Plant Sci. 2019 Nov 15;10:1445. doi: 10.3389/fpls.2019.01445 (PMC6873630; doi:10.3389/fpls.2019.01445)
Supplement: Supplementary file 1 [file Presentation_1.pptx]

## Slide 1
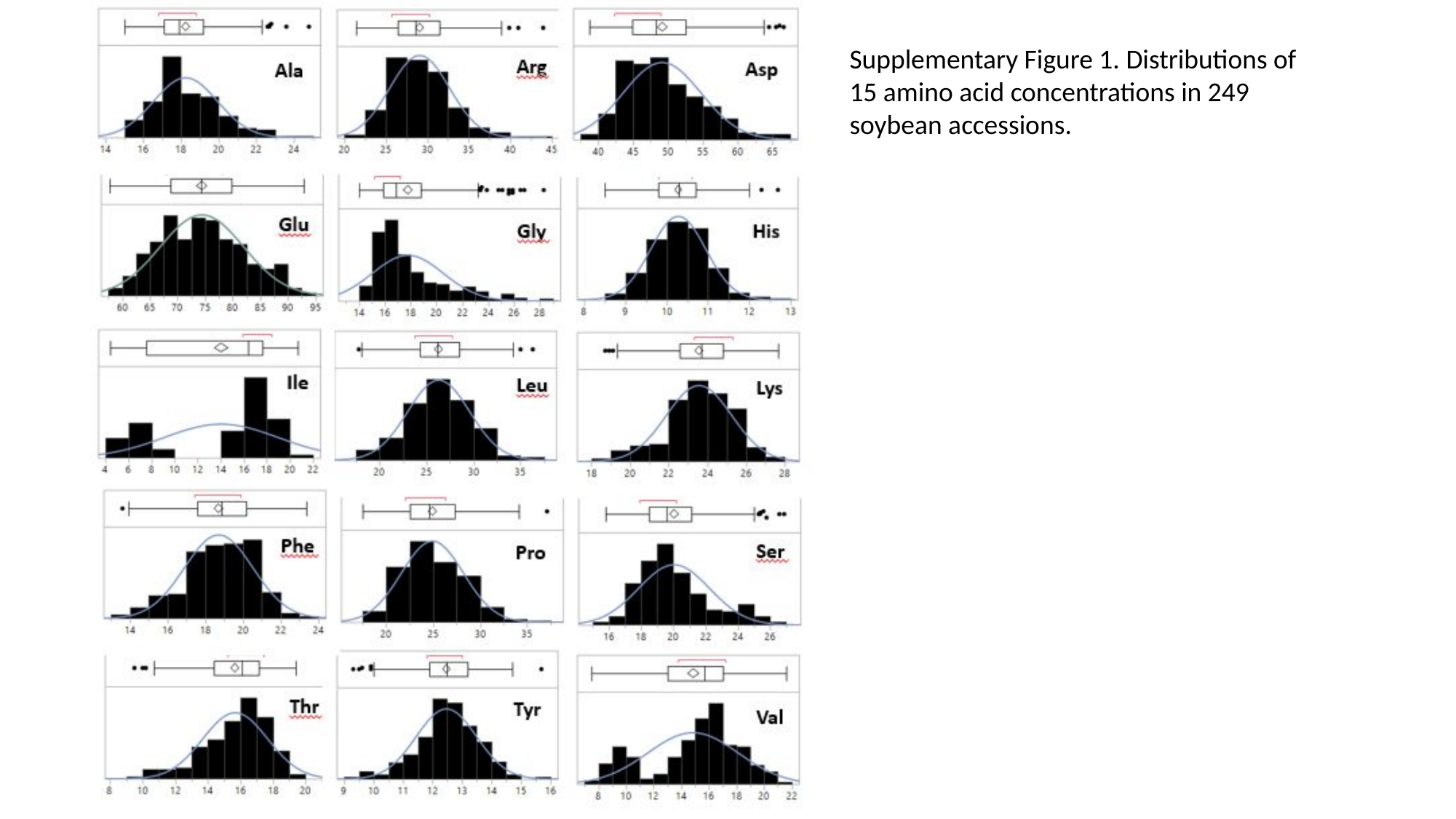

Supplementary Figure 1. Distributions of 15 amino acid concentrations in 249 soybean accessions.

## Slide 2
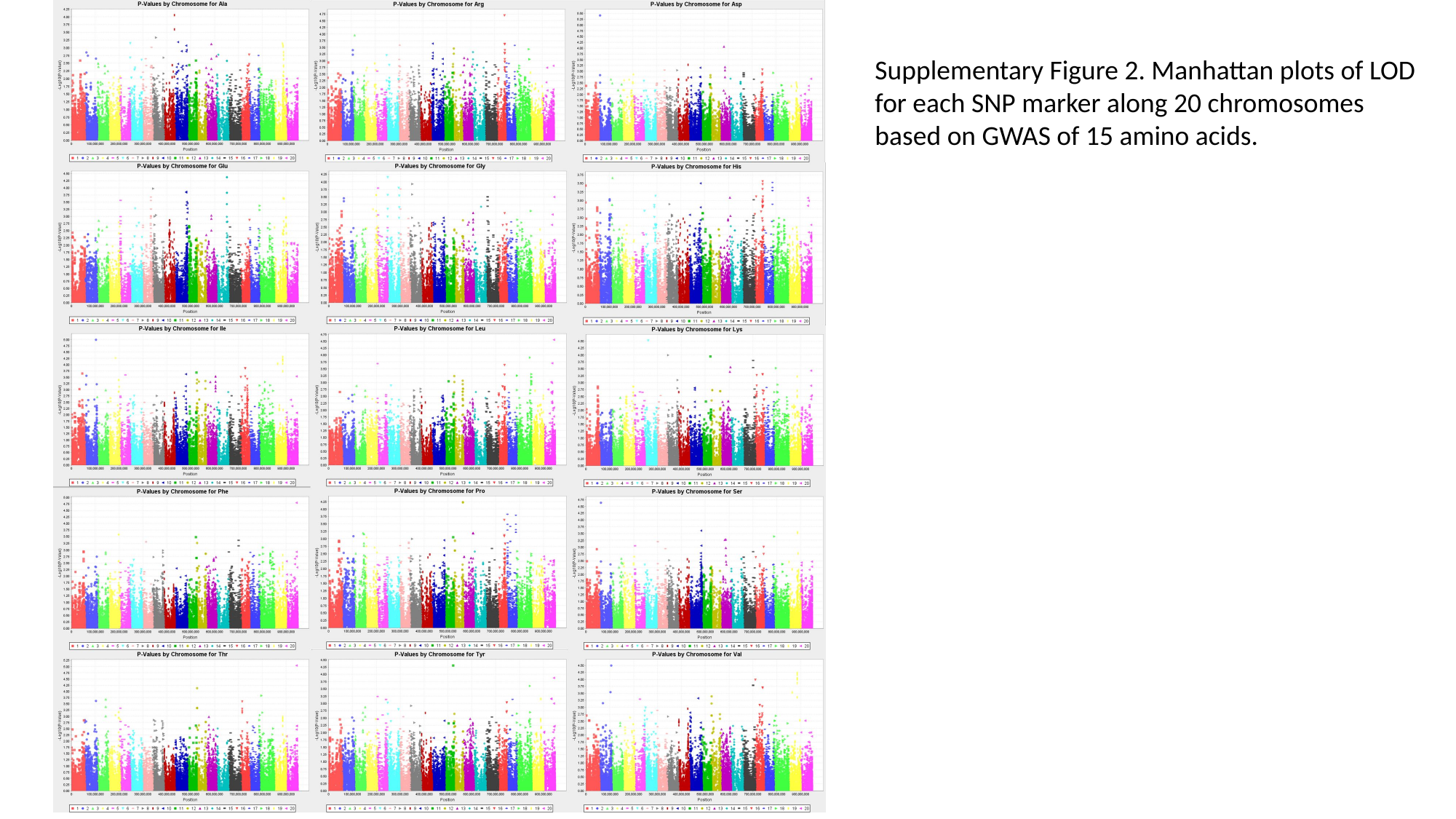

Supplementary Figure 2. Manhattan plots of LOD for each SNP marker along 20 chromosomes based on GWAS of 15 amino acids.

## Slide 3
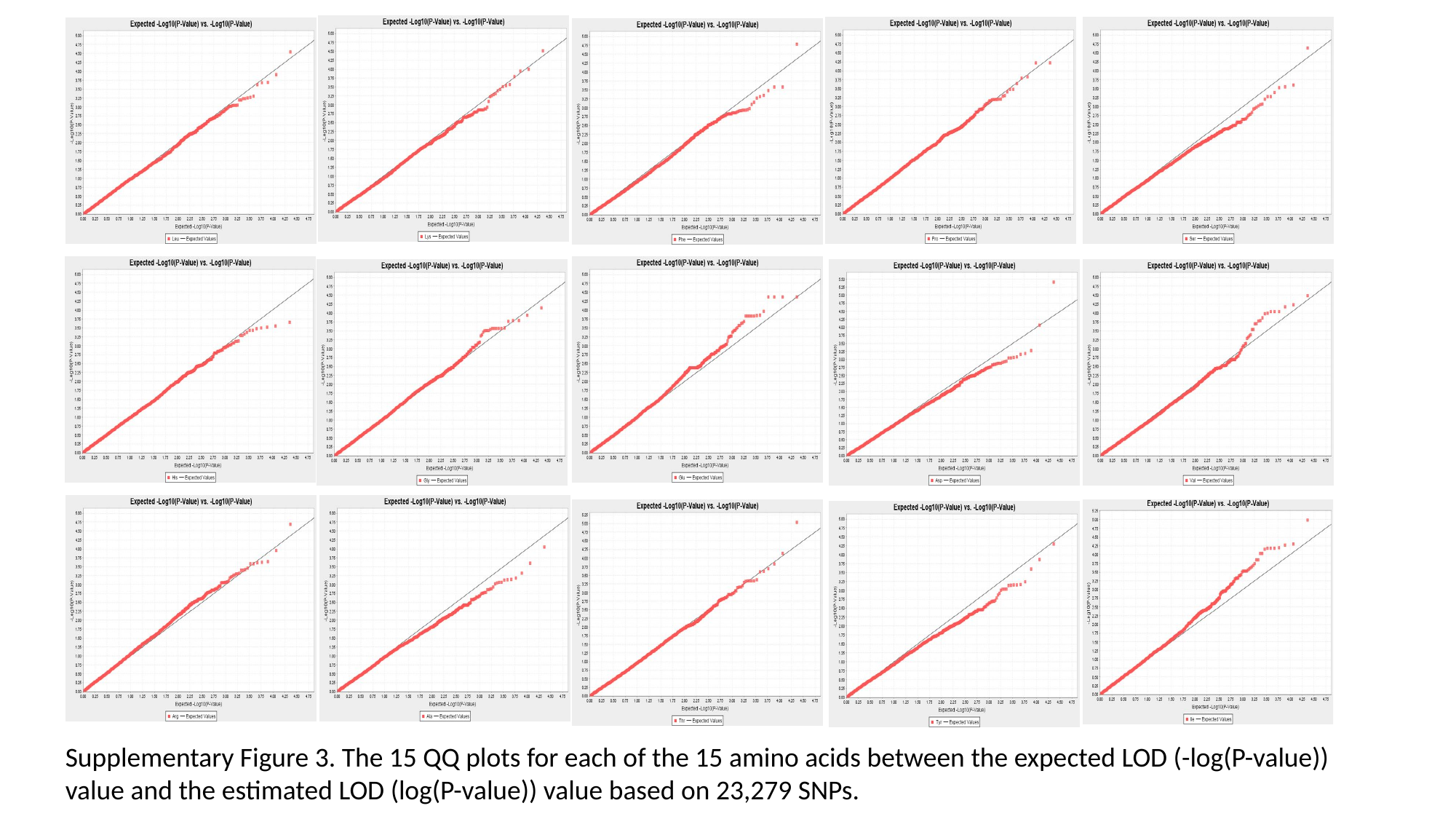

Supplementary Figure 3. The 15 QQ plots for each of the 15 amino acids between the expected LOD (-log(P-value)) value and the estimated LOD (log(P-value)) value based on 23,279 SNPs.
